# Socio-economic and health determinants of preference for separate living among older adults: A cross-sectional study in India

**DOI:** 10.1371/journal.pone.0249828

**Published:** 2021-04-14

**Authors:** T. Muhammad, Arun Balachandran, Shobhit Srivastava

**Affiliations:** 1 Department of Population Policies and Programs, International Institute for Population Sciences, Mumbai, Maharashtra, India; 2 Department of Sociology, University of Maryland, College Park, MD, United States of America; 3 Department of Mathematical Demography and Statistics, International Institute for Population Sciences, Mumbai, Maharashtra, India; Texas Technical University Health Sciences Center, UNITED STATES

## Abstract

**Introduction:**

The living arrangements among the older population form a basic pointer to the care and support of older adults in India, and living with extended kin is clearly differentiated from living separately. This paper attempts to understand the associations between socio-economic and health-related variables with preference for the separate living among older adults in India.

**Materials and methods:**

Using data from Building Knowledge Base on Population Ageing in India (BKPAI), we employed bivariate and probit regressions on a sample of 9540 older adults to fulfil the study objective.

**Results:**

Nearly 21% of older adults were living alone/with a spouse. Additionally, those older adults who lived alone/with spouse had specific reasons, i.e. about 14.6% reported that they had no children, 47.3% of older adults had their children away and 15.9% of older adults reported a family conflict. Availability of children is consistently found to be negatively associated with the preference of separate living. Besides, better self-rated health, independence in daily activities, and facing any type of violence were the strongest predictors of preference for separate living. In addition, the background characteristics, including age, sex, education, religion, and ethnicity, were found as significant predictors of living arrangement preference. Preference for co-residential arrangements emerges among older persons who have a feeling of importance within their family.

**Conclusion:**

Physical proximity to kin and health conditions, in addition to economic conditions, substantially determine the swing towards separate living among older adults in India. This suggests that attention has to be paid to the demand for specialized care and health services among older adults living separately.

## Introduction

The living arrangements of the older population are often considered as the basic indicator of the care and support provided by the family (Dostie & Thomas, 2004). A plethora of evidence from the developing world, especially in Asia, suggests that the family is the critical institution for older adults and their living arrangement is a fundamental determinant of well-being [1–3]. However, with modernization, joint living that is prevalent in traditional agricultural societies would become less common as industrialization and division of labor increases [4].

Much of the discussion on living arrangements among older adults in India concludes that social changes resulted in the traditional family-based care to be less the norm and there is a gradual decline of the joint family [5–7], dominated by a nuclear family setup wherein the older adults reside independently of their children or in other living facilities. Studies dealing with the living arrangement choices of the older adults indicated that most of, the older adults preferred co-residence while a substantial proportion also preferred to live alone or with a spouse [8–10]. With an increase in age, the older adults’ joint responsibility for support, care and upbringing of children decreases and they gradually withdraw from economic activities. Eventually, the aged generally become dependent upon their grown-up children [11]. Whereas the primary responsibility of the multi-generational co-residence system has been to protect these dependent members of the family and to provide income, healthcare, and security [12]. This signifies the important role of the family and its structure on the lives of older individuals.

### Living arrangement preferences in old age

The demographic availability hypothesis suggests that the availability of adult children is an important determinant of living arrangements among older individuals [13–15]. On the other hand, it is demonstrated that rising incomes of older adults is also responsible for the increase in the proportion of solo living among older population. In the purchase of privacy hypothesis, based on an economics perspective, Michael et al. (1980) argue that income level is the primary factor that determines the propensity to live alone, which they see as a reflection of economic demand for privacy [16]. It is also revealed that independent living is a normal good, which is purchased more as a person’s income rises [17]. Since economic status is a major factor determining the living arrangement choices, it has also been proven to be true in the context of developing countries as well [4, 18–21].

Further, a couple of studies revealed that older women who are more likely to survive to older ages in comparison to their men counterparts [22], prefer living alone or with spouse than other living arrangements [23, 24]. A few studies in India also report an increase of widowed older women resulting in a striking number of women who live alone [10]. Additionally, as it is evident from multiple studies that privacy is a major determinant of living arrangements the divorcees may have been prepared to live independently and will be more comfortable living alone [25, 26].

Though descriptive studies on the patterns and changes of living arrangements are not entirely novel in the Indian context [5, 9, 10, 27, 28], the key factors that determine residential preferences of older adults are least addressed in the research. In this backdrop, the paper attempts to understand the socio-economic and health determinants of preference for the separate living among older adults. An attempt is made to explain why do they prefer to break away from the norms of jointly living and are asserting their need for privacy and autonomy. The study also broadly reflects on greater details into the increase in separate living arrangements among older adults in India.

## Material and methods

### Data source

The present research used data from Building Knowledge Base on Population Ageing in India (BKPAI), which was a national level survey and was conducted in 2011 across seven states of India [29]. The survey was implemented by UNFPA along with research institutes in India- the Institute for Social and Economic Change (ISEC), Tata Institute for Social Sciences (TISS), and Institute for Economic Growth (IEG). The survey gathered information on various socio-economic and health aspects of aging among households of those aged 60 years and above. Seven major regionally representative states, with the highest 60+ years population than the national average, were selected for the survey. This survey was carried out on a representative sample in the Northern, Western, Eastern, And Southern parts of India following a random sampling process [29].

The primary sampling unit (PSU) were villages for rural areas and urban wards in urban areas. The sample of 1280 older adult households was fixed for each state. Further details on the sampling procedure, the sample size is available in national and state reports of BKPAI, 2011 [29]. For the current study, a sample size of 9540 older adults residing in seven states aged 60+ years was selected.

### Variable descriptions

#### Outcome variable

The outcome variable is a binary classification of the living arrangement. It was formed using the question- ‘What is your preferred living arrangement?’, which had responses of ‘alone’, ‘with spouse only’, ‘with sons’, ‘with daughters’, ‘either sons or daughters’, ‘with other relatives’, ‘in an old age home’ and ‘other’. It was then recoded as 0, for responses of ‘with sons’, ‘with daughters’, ‘either sons or daughters’, ‘with other relatives’ and 1 for ‘alone’, ‘with spouse only’ [30].

#### Predictor variable

The explanatory variables were categorized into three sections, namely background factors, economic condition, and health status. Background factors were as age categorized as (60–69, 70–79 and 80+), sex was categorized as (men and women), marital status was categorised as (not in a union and currently in a union), educational status was categorized as (none, below 5 years, 6–10 years, 11+ years), the residence was categorized as (rural and urban), religion was categorised as (Hindu, Muslim, Sikh, and others), caste was categorized as (Scheduled Caste, Scheduled Tribe, Other Backward Class, and others), children above born was categorized as (no child, single child, two children, and three or more children), decision making was categorized as (no role, partial decision making and absolute) and importance of family was categoriszd as (important and somewhat/not important). Economic conditions were as assets ownership categorized as (0 “no asset owned”, 1 and 2+), working status was categorized as (unemployed and employed), money contributed in household expenditure (not contributed and contributed). Health status was as violence categorized as (no and yes). Self-rated health (SRH) was categorized as (very good “very good/excellent”, good “good” and poor “fair/poor”) [31]. Ability to do activities of daily living (ADL) was having a scale of 0 to 6 wherein it represents higher the score higher the independence [32]. A score was categorized as 0, which represents full independence, and five and less were categorized as 1, which represents not fully independent to do activities of daily living (Cronbach alpha: 0.89) [33–35]. The ability to do instrumental activities of daily living (IADL) was having a scale of 0 to 8, representing higher the score higher the independence [36]. A score of 6+ was categorized as 0 representing high IADL and a score of 5 and less was recoded as 1 representing low IADL [36, 37]. States of India where the survey was conducted as (Himachal Pradesh, Punjab, West Bengal, Orissa, Maharashtra, Kerala, Tamil Nadu).

### Statistical analysis

Bivariate analysis was done to find the unadjusted association between the preferred separate living and other predictor variables for older adults in India. To find the adjusted association, probit regression analysis was used in the study. It uses to model dichotomous or binary outcome variables. In the probit model, the inverse standard normal distribution of the probability is modelled as a linear combination of the predictors [38, 39].

In probit regression, the cumulative standard normal distribution function Φ(.) is used to model the regression function when the dependent variable is binary, that is, we assume

$$E(Y|X)=P(Y=1|X)=\Phi (\beta_{0}+\beta_{1}X)$$ (1)

*β*_0_+*β*_1_*X* in (1) plays the role of a quantile z. Remember that

Φ(z)=P(Z≤z),Z∼N(0,1)

such that the probit coefficient β1 in (1) is the change in z associated with a one-unit change in X. Although the effect on z of a change in X is linear, the link between z and the dependent variable Y is nonlinear since Φ is a nonlinear function of X.

Assume that Y is a binary variable. The model

$$Y=\beta_{0}+\beta_{1}+X_{1}+\beta_{1}\beta_{2}\ldots \ldots \ldots +\beta_{K}\beta_{K}+u$$

with

$$P(Y=1|X_{1},X_{2},\ldots \ldots .,X_{K})=\Phi (\beta_{0}+\beta_{1}+X_{1}+\beta_{1}\beta_{2}\ldots \ldots \ldots +\beta_{K}\beta_{K})$$

is the population Probit model with multiple regressors *X*_1_,*X*_2_,…….,*X_K_* and Φ(⋅)is the cumulative standard normal distribution function [40]

## Results

Table 1 represents the socio-economic profile of older adults in India. About 33% of older adults preferred separate living. Nearly 10.3% of older adults belong to the age group 80 years and above. Women constitute 52.5% of the total interviewed respondents. About 4 in 10 older adults were not in the union at the time of the interview. Only about 1 in 10 older adults were highly educated. Approximately 52.3% of an older adult was from an urban place of residence. Nearly 8 in 10 older adults were from the Hindu religion, and 4 in 10 older adults were from other caste categories. Almost 6.4% of older adults were not involved in any of the household activities, and 5% of older adults had no role in decision-making in the household. About 35% of older adults felt that they had somewhat or no importance in the family. About 5 in 10 older adults were dependent or having no income. 18% of older adults had no asset ownership, and 54% had 2 or more asset ownership. About 36% of older adults were unemployed in the last year. About 47% of older adults did not contribute money in the household expenditure (this also includes those older adults who do not have any income). About 1 in 10 older adults had to face violence. 53.5% had bad SRH, 7.4% had low ADL, and 53.7% had bad IADL. 14% of the older adult respondents belong to Kerala.

**Table 1:** Socio-economic profile of older adults in India. (N = 9540).

Variables	Categories	Percentage
***Outcome variable***		
**Preferred Separate living**	No *“preferred living with others”*	67.0
	Yes “*preferred living alone or with spouse*”	33.0
***Background variables***		
**Age group (years)**	60–70 “*60–69”*	63.3
	70–80 *“70–79”*	26.5
	80+ “*80 and above*”	10.3
**Sex**	Men	47.5
	Women	52.5
**Marital status**	Not in union “*never married*, *separated*, *divorced*, *widowed*”	40.4
	Currently in union *“currently married”*	59.6
**Years of schooling**	None	46.0
	below 5 years	20.5
	6–10 years	25.0
	11+ years	8.6
**Place of residence**	Rural	52.3
	Urban	47.7
**Religion**	Hindu	80.3
	Muslim	6.7
	Sikh	8.6
	Others “*other than Hindu*, *Muslim and Sikh”*	4.4
**Caste**	Scheduled Caste	19.7
	Scheduled Tribe	5.1
	Other Backward Class	35
	Others	40.3
**Children ever born**		
	No child	3.8
	Single child	9.1
	2 children	17.9
	3 and above	69.3
**Decision making**	No role “*not involved in any decision taken in the households”*	5.0
	Partial role “*involved in some decisions taken in the household*”	24.6
	Absolute role “*involved in every decision taken in the household”*	70.4
**Importance to family**	Important	65.1
	Somewhat/not important	34.9
***Economic condition***		
**Asset ownership**	0 “*No asset ownership*”	18.0
	1 “*one asset owned*”	27.8
	2+ “*at least two assets owned*”	54.1
**Working status**	Unemployed	36.1
	Employed	63.9
**Money contributed in household expenditure**	Contributed “*had income and contributed too*”	52.8
	Not contributed “*had income and not contributed/ no income*”	47.2
***Health status***		
**Violence**	No “*not faced violence/abuse/neglect or disrespect*”	90.1
	Yes “*faced violence/abuse/neglect or disrespect”*	9.9
**SRH**	Very good “*very good and excellent*”	16.6
	Good “*good*”	29.9
	Poor “*poor or fair*”	53.5
**ADL**	High “score of *6+*”	92.6
	Low “score of 0 to 5”	7.4
**IADL**	High “score of *6+*”	46.3
	Low “score of 0 to 5”	53.7
**States**	Himachal Pradesh	15.5
	Punjab	14.2
	West Bengal	11.7
	Orissa	15.2
	Maharashtra	14.5
	Kerala	14.2
	Tamil Nadu	14.8

*SRH*: *Self-rated health; ADL*: *Activities of daily living; IADL*: *Instrumental activities of daily living*.

**Fig 1:**
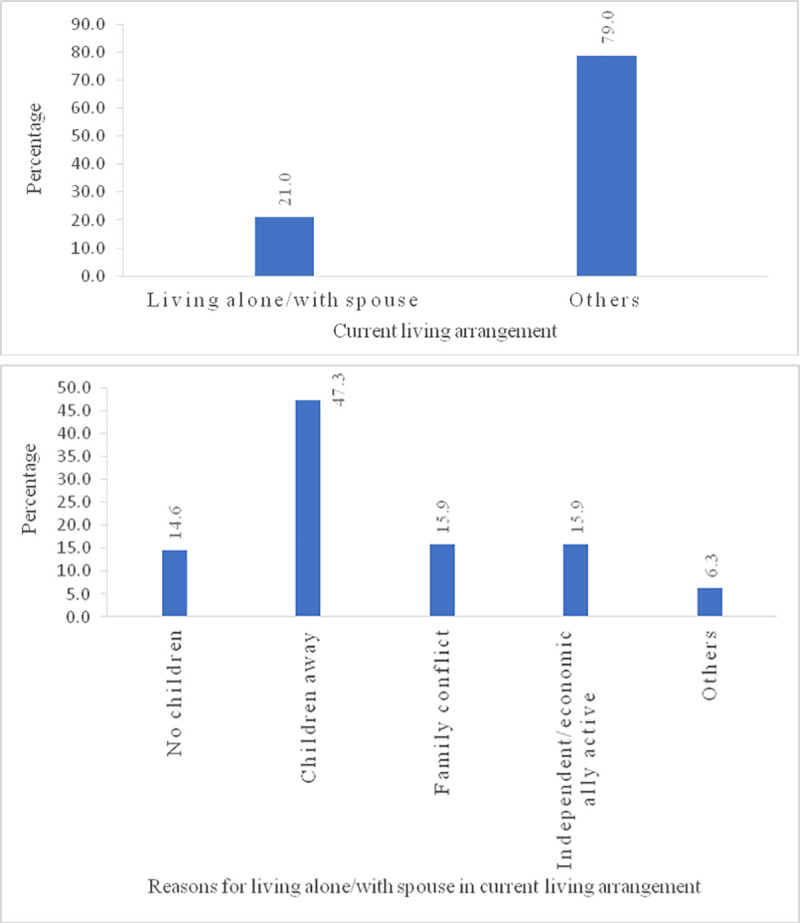
A. Percentage distribution of current living arrangement among older adults in India. B. Percentage distribution of reasons for living alone/ with spouse in current living arrangement.

Fig 1A and 1B represent the percentage of living alone/with spouse and reason for living alone/with a spouse. Nearly 21% of older adults were living alone/with a spouse. Additionally, those older adults who lived alone/with spouse had giver specific reasons, i.e. about 14.6% told that they had no children, 47.3% of older adults said that they had their children away and 15.9% of older adults said they live alone/with a spouse because they had a family conflict.

Table 2 presents the bivariate association between older adults preferring to live separately over different background variables. We are not discussing these estimates and will primarily focus on adjusted estimates in Table 3 as they provide precise estimates as controlled for background factors.

**Table 2:** Bivariate association between older adults preference over living in private by different background factors in India (N = 9540).

Variables	Categories	Total	Men	Women
*Background variables*		%	%	%
**Sex**	Men	37.6		
	Women	30.4		
**Age group (years)**	60–70	34.3	36.7	32.1
	70–80	33.8	38.9	29.2
	80+	31.3	40.0	23.5
**Marital status**	Not in union	21.6	24.0	21.1
	Currently in union	41.8	40.2	44.8
**Years of schooling**	None	35.0	41.1	32.1
	below 5 years	30.2	33.5	26.5
	6–10 years	31.2	34,0	24.9
	11+ years	45.6	47.1	41.2
**Place of residence**	Rural	36.1	39.8	32.6
	Urban	27.5	31.1	24.4
**Religion**	Hindu	33.7	37.5	30.2
	Muslim	24.4	26.1	23.2
	Sikh	44.5	49.6	39.7
	Others	28.4	30.7	26.5
**Caste**	Scheduled Caste	40	45.7	34.6
	Scheduled Tribe	25.8	28.7	23.5
	Other Backward Class	31.5	33.7	29.6
	Others	33.9	38.2	30
**Children ever born**	No child	54.3	58.7	50.7
	Single child	28.3	34.2	23.9
	2 children	36.2	39.7	32.0
	3 and above	32.8	36.3	29.7
**Decision making**	No role	32.8	45.1	29
	Partial role	26	30.3	22.8
	Absolute role	36.6	39.4	33.6
**Importance to family**	Important	33.1	36.2	29.6
	Somewhat/not important	35.1	41.2	31.4
***Economic condition***				
**Asset ownership**	0 “No asset ownership”	41.4	43	41
	1	34.2	42.4	28.2
	2+	31.2	35.5	24.8
**Working status**	Unemployed	29	42.5	28.8
	Employed	36.5	37.6	33.7
**Money contributed in household expenditure**	Contributed	36.5	40.2	29.6
	Not contributed	30.9	31.2	30.8
***Health status***				
**Violence**	No	33.5	37.6	30.2
	Yes	34.6	37.9	31.8
**SRH**	Very good	38.5	41.6	34.6
	Good	36.1	38.6	33.7
	Poor	31.4	35.7	27.9
**ADL**	High	34.5	37.9	31.3
	Low	26	33.8	21.3
**IADL**	High	38.4	41.3	35.7
	Low	30.3	34.7	26.4
**States**	Himachal Pradesh	52.7	56.1	49.4
	Punjab	46.2	50.9	41.8
	West Bengal	24.8	31.4	18.8
	Orissa	14.2	16.8	11.5
	Maharashtra	23.7	24.1	23.3
	Kerala	22.5	27.5	18.8
	Tamil Nadu	50.8	55.2	46.9

*SRH*: *Self-rated health; ADL*: *Activities of daily living; IADL*: *Instrumental activities of daily living*.

**Table 3:** Probit regression analysis for older adults preference over living in private by different background factors in India.

Variables	Categories	Total Sample (N = 9540)	Men (N = 4531)	Women (N = 5009)
		Coef. (95% CI)	Coef. (95% CI)	Coef. (95% CI)
***Background variables***				
**Sex** *Ref*: *Men*	Women	0.09(-0.01,0.18)		
**Age group (years)** *Ref*: *60–70*	70–80	0.06(-0.01,0.13)	0.09(0,0.19)	0.02(-0.08,0.12)
	80+	0.05(-0.06,0.16)	0.18*(0.03,0.33)	-0.07(-0.22,0.09)
**Marital status** *Ref*: *Not in union*	Currently in union	0.70*(0.62,0.77)	0.62*(0.5,0.74)	0.72*(0.62,0.81)
**Years of schooling** *Ref*: *None*	Below 5 years	-0.03(-0.12,0.05)	-0.02(-0.14,0.1)	-0.05(-0.16,0.07)
	6–10 years	-0.10*(-0.19,-0.01)	-0.10(-0.22,0.01)	-0.08(-0.21,0.06)
	11+ years	0.25*(0.12,0.38)	0.21*(0.04,0.37)	0.42*(0.19,0.65)
**Place of residence** *Ref*: *Rural*	Urban	-0.18*(-0.24,-0.12)	-0.18*(-0.27,-0.09)	-0.17*(-0.26,-0.08)
**Religion** *Ref*: *Hindu*	Muslim	-0.03(-0.16,0.09)	-0.14(-0.33,0.04)	0.1(-0.08,0.27)
	Sikh	-0.15*(-0.28,-0.02)	-0.09(-0.28,0.1)	-0.21*(-0.4,-0.02)
	Others	0.06(-0.09,0.21)	-0.05(-0.27,0.16)	0.15(-0.06,0.36)
**Caste** *Ref*: *Scheduled Caste*	Scheduled Tribe	-0.06(-0.21,0.1)	-0.09(-0.3,0.13)	-0.03(-0.25,0.2)
	Other Backward Class	-0.19*(-0.28,-0.1)	-0.24*(-0.37,-0.11)	-0.15*(-0.28,-0.03)
	Others	-0.09*(-0.17,0)	-0.14*(-0.26,-0.03)	-0.04(-0.16,0.08)
**Children ever born** Ref: No child	Single child	-.68* (-0.84, -0.51)	-0.67*(-0.92, -0.42)	-0.66* (-0.89, -0.43)
	2 children	-.60*(-0.75, -0.45)	-0.61*(-0.84, -0.38)	-0.57* (-0.78, -0.36)
	3 and above	-.73*(-0.88. -0.59)	-0.75*(-0.97, -0.53)	-0.70*(-0.90, -0.51)
**Decision making** *Ref*: *No role*	Partial role	-0.09(-0.25,0.07)	-0.06(-0.37,0.24)	-0.10(-0.29,0.09)
	Absolute role	0.13(-0.03,0.28)	0.12(-0.18,0.41)	0.16(-0.02,0.35)
**Importance to family** *Ref*: *Important*	Somewhat/not important	0.19*(0.12,0.25)	0.16*(0.06,0.25)	0.21*(0.12,0.3)
***Economic conditions***				
**Asset ownership** *Ref*: *0 “No asset ownership”*	1	-0.09(-0.17,0)	-0.06(-0.23,0.1)	-0.1(-0.21,0.01)
	2+	-0.22*(-0.31,-0.13)	-0.20*(-0.36,-0.04)	-0.22*(-0.33,-0.11)
**Working status** *Ref*: *Unemployed*	Employed	0.11*(0.02,0.20)	-0.13(-0.44, 0.18)	0.13* (0.13, 0.24)
**Money contributed in household expenditure** *Ref*: *Not Contributed*	Contributed	0.22*(0.11,0.33)	0.38*(0.21,0.55)	0.12(-0.03,0.28)
***Health status***				
**Violence** *Ref*: *No*	Yes	0.26*(0.16,0.36)	0.20*(0.05,0.34)	0.31*(0.17,0.45)
**SRH** *Ref*: *Very Good*	Good	0.01(-0.09,0.09)	0.01(-0.11,0.13)	0.01(-0.13,0.14)
	Poor	-0.12*(-0.21,-0.03)	-0.11(-0.23,0.01)	-0.13(-0.25,0)
**ADL** *Ref*: *High*	Low	-0.08(-0.2,0.04)	-0.02(-0.21,0.17)	-0.10(-0.27,0.06)
**IADL** *Ref*: *High*	Low	-0.29*(-0.35,-0.22)	-0.30*(-0.39,-0.21)	-0.28*(-0.37,-0.18)
**States** *Ref*: *Himachal Pradesh*	Punjab	-0.12(-0.25,0)	-0.08(-0.26,0.1)	-0.18*(-0.36,-0.01)
	West Bengal	-0.62*(-0.74,-0.51)	-0.52*(-0.68,-0.37)	-0.73*(-0.9,-0.56)
	Orissa	-1.24*(-1.36,-1.12)	-1.2*(-1.36,-1.04)	-1.3*(-1.48,-1.13)
	Maharashtra	-0.89*(-1.01,-0.78)	-0.87*(-1.03,-0.71)	-0.94*(-1.11,-0.77)
	Kerala	-0.65*(-0.77,-0.53)	-0.52*(-0.69,-0.35)	-0.78*(-0.95,-0.6)
	Tamil Nadu	0.17*(0.05,0.3)	0.21*(0.04,0.39)	0.15(-0.03,0.32)

*Ref*: References; *if p<0.*05; CI*: *Confidence Interval; SRH*: *Self-rated health; ADL*: *Activities of daily living; IADL*: *Instrumental activities of daily living*.

Table 3 represents the probit estimates for older adults who preferred separate living. Oldest-old men had a higher probability of preferred living separate than older adults from the young-old age group [Coef. 0.18, p<0.05]. The older adults including men and women who were currently in the union had a higher probability of preferring living separate than older adults not in union (Total [Coef. 0.70, p<0.05], Men [Coef. 0.62, p<0.05]. Women [Coef. 0.72, p<0.05].) Older adults including men and women who had years of schooling 11 years and above had a higher probability of preferring lining separate than older had no educational status (Total [Coef. 0.25, p<0.05], Men [Coef. 0.21, p<0.05] and Women [Coef. 0.42, p<0.05]). The probability of preferring to live separately was low among urban residents than rural ones (Total [Coef. -0.18, p<0.05], Men [Coef. -0.18, p<0.05], and Women [Coef. -0.17, p<0.05]).

Older adults who were having absolute involvement in household activities had a lower probability of preferring living separate than older adults who had no involvement in household activities (Total [Coef. 0.25, p<0.05], Men [Coef. 0.21, p<0.05], and Women [Coef. 0.42, p<0.05]). The probability of preferring separate living was higher among older adults who had absolute involvement in decision making than those who had no role in decision making; however, the result is not significant. (Total [Coef. 0.13, p>0.05], Men [Coef. 0.12, p>0.05] and Women [Coef. 0.16, p>0.05]). Older adults who feel their family give somewhat or no importance to them had a higher probability of preferring living separate than those whose family members give importance to them (Total [Coef. 0.19, p<0.05], Men [Coef. 0.16, p<0.05] and Women [Coef. 0.21, p<0.05]). Asset ownership played a protective role in for settlement of older adults separately, i.e. older adults owning two or more assets had a lower probability of preferring for living separate than older adults who do not own any asset (Total [Coef. -0.22, p<0.05], Men [Coef. -0.20, p<0.05] and Women [Coef. -0.22, p<0.05]). Older adults who were employed had a higher probability of preferring to live separately than older adults who were unemployed (Total [Coef. 0.11, p<0.05] and Women [Coef. 0.013, p<0.05]). The probability of preferring living separate was higher among older adults who contribute money in the household expenditures/budget than those who did not contribute money in the household expenditure/budget (Total [Coef. 0.22, p<0.05], Men [Coef. 0.38, p<0.05] and Women [Coef. 0.12, p>0.05]).

Older adults who faced any type of violence had higher probability to prefer living separate than older adults who did not face any type of violence (Total [Coef. 0.26, p<0.05], Men [Coef. 0.20, p<0.05] and Women [Coef. 0.31, p<0.05]). Probability to prefer living separate was low among older adults who had poor SRH (Total [Coef. -0.12, p<0.05], Men [Coef. -0.11, p>0.05] and Women [Coef. -0.13, p>0.05]), low ADL (Total [Coef. -0.08, p>0.05], Men [Coef. -0.02, p>0.05] and Women [Coef. -0.10, p>0.05]) and low IADL (Total [Coef. -0.29, p<0.05], Men [Coef. -0.30, p<0.05] and Women [Coef. -0.28, p<0.05]) than those older adults who had very good SRH, high ADL and high IADL. Older adults from Tamil Nadu had higher probability to prefer living separate than older adults from Himachal Pradesh (Total [Coef. 0.17, p<0.05], Men [Coef. 0.21, p<0.05] and Women [Coef. 0.15, p>0.05]).

## Discussion

The analysis of current living arrangements suggests that although co-residence is the major form of living arrangement, separate living is also on the rise among older adults in India. This is in a backdrop of a strong filial obligation rooted in intergenerational co-residence in India, like many other Asian countries [41–43]. It is perhaps quite true that older adults in India are traditionally taken care of by the family, and this accepted norm is also shown in their preferences as the majority still prefer to live with their family rather than to stay alone [44]. However, the Indian system of old-age support is more complex than the norm suggests and the preferences are in transition [43].

Almost all the older adults who preferred to live alone or with spouse did so mainly because of the lack of availability of adult children in their proximity. This points at how the older Indian adult still looks up to their adult children for intergenerational support and how a separate living is preferred by them in the absence of support from adult children [45]. The other important reasons for preferring separate living were family conflict, the need for independence or privacy, and better economic conditions. On the other hand, the analysis of living arrangement preferences reveals that most of the background variables are negatively associated with preferring separate residences. However, the study found a positive association between living in urban areas and preferring co-residence which is contradictory to the conventional assumption that urbanization, alongside exposure to Western or individualistic values, has increased the number of nuclear households and preference of the young to live separately from the older generation [42].

Our study is in agreement with previous literature in the Asian context. A study based on the data from Beijing has shown that married older adults were less likely to co-reside with their children [46]. Another study found that marital status was a significant predictor of living alone, where widowed were more likely to live alone compared to their married counterparts [47]. In agreement with such studies, the results of the present study show that the older adults who are currently in a marital union prefer separate living suggesting that marital status is a significant factor in determining living arrangement choices among both men and women alike. Compared to currently married, unmarried older adults are more likely to seek companionship from living in alternative arrangements. Education is another factor that affects their living preference. It was found that the respondents with formal education (above 10 years of schooling) have higher probabilities of living in their own houses compared to those who have no education or up to 10 years of schooling. This finding can be explained as individuals with a low level of education are less likely to have the ability to make household decisions [48]. They need support from their children and immediate family members to live in their old age. Based on the probit model, the education effect is significant for both men and women.

Our results that indicate that the preference of living arrangement is shaped by individual-level factors as well as household characteristics confirm earlier research conducted in India [10, 44]. Strikingly it was found that the older adults who realize their importance in the household, prefer to co-reside with their family or relatives. Thus, the increasing trend of separate living preference among older Indian adults could be explained by the social changes that disrupted the co-residential arrangements, and the gradual decline in the respect accorded to the older people in South Asian cultures [30, 42, 49]. At the same time, the results also showed that a partial or absolute role of older adults in the household decision-making are encouraged to prefer living alone or with the spouse who may provide them a sense of autonomy [21]. Meanwhile, studies found that the older adults who moved from the centers to the peripheries for living took on fewer domestic duties and gained respect but with a loss of power and authority [50].

Consistent with the demographic availability hypothesis [13–15], in the present study, the number of living children acts as an indicator of co-residence. It is found that the probability of preferring separate living is related positively to the number of adult children available to older adults. Further, Wister (1984) suggested that the rise in real income has allowed for the increased purchase of privacy among older adults [15]. The present study found that the likelihood of preferring separate residence is higher among the older adults who contributed their economic share in the household expenditure. The positive relationship between the working status of the older adults and their independent living preference was also found significant.

Contrary to this, the economic status of older adults indicated by ownership of dwelling unit and the land either self-acquired or inherited to which they are financially independent of their children suggests that it is negatively associated with the choice of separate living and those with economic resources prefer to live on their own and do not want to get apart. Unlike assets belonging to the household, personal assets are the resources that the older adults can fall back on in times of need and which give them a feeling of power and dignity [51]. It also suggests that such ownership endows the older adults in India with symbolic and cultural status within the household as well as in society [52, 53].

It was found that living in a separate residence seems the desired option for older adults in good health. It reveals that those in better health are more likely than those in poor health to prefer separate arrangements. Surprisingly, we do not find it significant among men and women, which may imply that this is a weak objective measure of health among older adults in developing countries. Hence, further study is warranted. Besides, the selection of health measures was also based on the significance of physical mobility as it relates to daily coping ability. The results of ADL and IADL as the ability to handle tasks that are integral for day-to-day living imply that, if they can, the older parents want to avoid burdening other members of the household through co-residence. On the other hand, the older adults who reported a higher level of dependency on others in ADL or higher limitation in the instrumental ADL preferred not to live separately indicating that the older adults depend on children for care because of deteriorating health as they grow older [18]. Further, those who faced any type of violence, which includes verbal, financial, physical, and emotional abuse or neglect and ignorance from the family members are more likely to desire separate living.

Nevertheless, the findings drawn from the results are only a product of cross-sectional data taken at one point in time. And the generalizations made from the present study to a larger population need to be done with caution. Older adults children’s educational status is one of the important factor for separate living among older adults in India. However, this information is not available in the dataset, therefore, the association cannot be estimated.

## Conclusions

The findings broadly suggest that a range of individual characteristics, including age, gender, marital status, physical health, and education have significant effects on the separate living preference of older adults. Further, the health and economic factors were found as significant predictors of the older adults preferring a separate living arrangement.

It is observed that an overwhelming majority of older adults who lack primary education, not in any employment, currently not in union prefer to co-reside. A positive interpretation of this would be that the co-residential arrangements are favourable for their overall well-being since co-residence with adult children is a reliable source of assistance and support [46]. Analysis of the preference for separate living by respondents shows that a large proportion of older adults desire to live alone or with a spouse in their better health and economically active status. However, poor SRH, higher limitation on IADL, and facing any type of violence surface as the strongest predictors of preference of living alone or with a spouse. Furthermore, many of the findings could also suggest the recent changes in the existing value systems in society.

Finally, the swing towards separate living has resulted in the family life of the older adults being highly influenced by physical proximity to kin in addition to a health condition and economic resources. This suggests attention to the demand for specialized services provided that allow older adults to remain in their homes for as long as possible in the face of other constraints, such as limited functional capacity. The living preferences should be coupled with respect for the individual’s autonomy and dignity within the family to enhance personal satisfaction. Further research is required, in particular, to promote a better understanding of pathways on decisions for separate living, and more specifically, to identify the factors involved in preferring separate living arrangements in older ages.
